# Supplementary material for: Molecular Oscillator Affects Susceptibility of Caterpillars to Insecticides: Studies on the Egyptian Cotton Leaf Worm—Spodoptera littoralis (Lepidoptera: Noctuidae)
Source: Insects. 2022 May 23;13(5):488. doi: 10.3390/insects13050488 (PMC9147166; doi:10.3390/insects13050488)
Supplement: Supplementary file 1 [file insects-13-00488-s001.zip › Figure S1 ost.pdf]

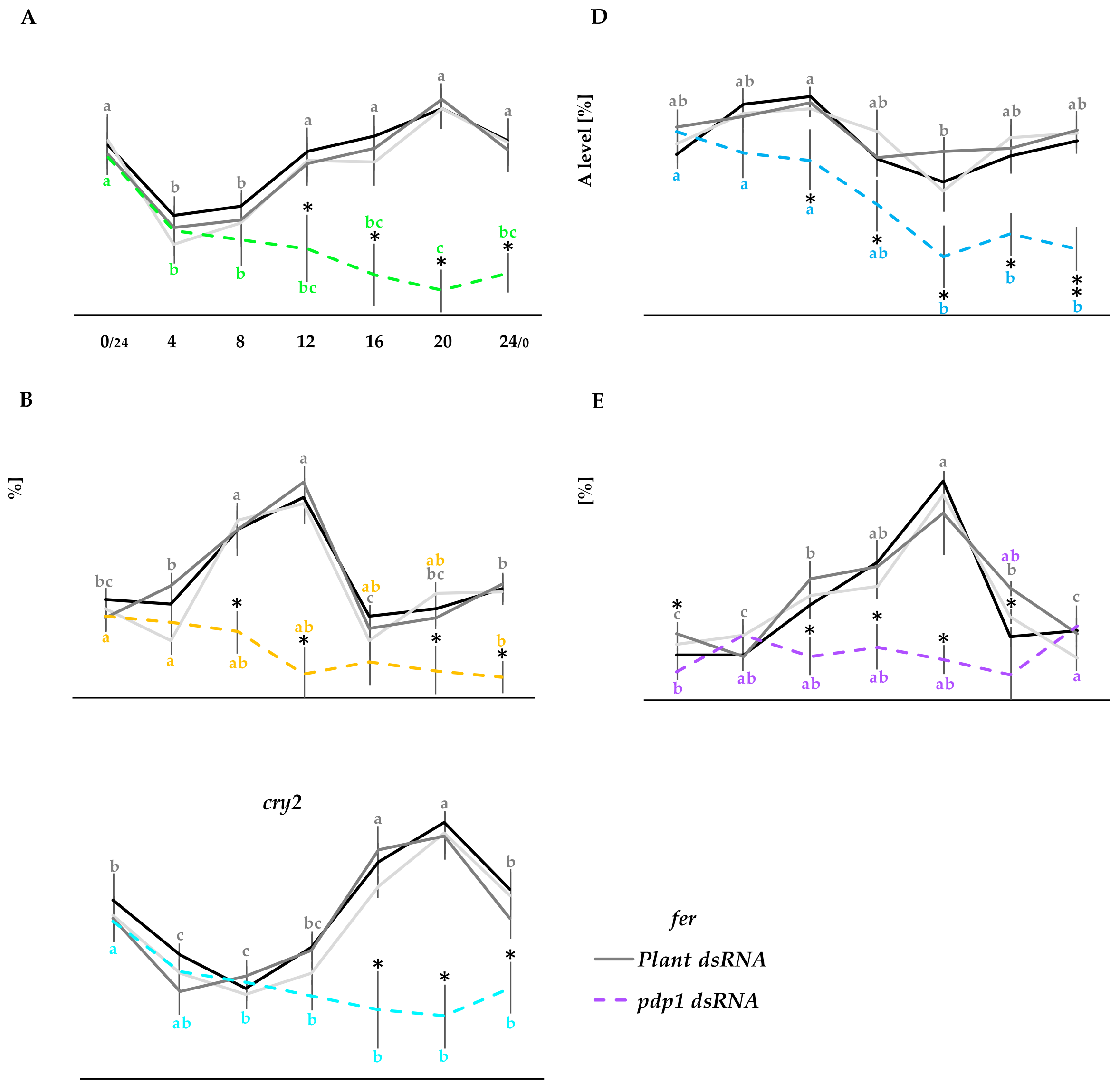

Figure S1. RNAi-mediated disruption of daily patterns of clock genes expression in the fat body. The dsRNA measuring approximately 300 bp was produced by *in vitro* transcription based on the coding fragments of clock genes. Then, 400 ng per larvae in transcription buffer was injected into the body cavity of 2-day-old 6th instar larvae reared in LD conditions. The level of clock gene mRNA was then determined in the tissues after 24 h starting from the time of injection (Zt 0). Graphs represent daily changes of *per* (A), *tim* (B), *cry2* (C), *cyc* (D), and *pdp1* (E) mRNA levels in larvae injected with clock gene dsRNAs (dashed color lines; green for *per*, orange for *tim*, turquoise for *cry2*, light blue for *cyc*, and purple for *pdp1*) and in larvae which serve as controls, that were untreated (Intact – black lines), injected with buffer used for *in vitro* transcription (Buffer – light grey lines) or *SSU* dsRNA of *Nicotiana tabacum* (Plant dsRNA – dark grey lines). Expression of genes was determined by qPCR. Efficiency-corrected, normalized, relative gene expression values averaged from three separate experiments are shown for each time point of each experimental variant. Values with different letters for data of the Intact group differ significantly ($p < 0.05$), as determined by an ANOVA and Bonferonni post-test. Significant differences between mean values for larvae treated with

clock gene dsRNA and those of control groups at a given time point was calculated by an ANOVA and unpaired t-test; here shown comparisons are these between the groups treated with the dsRNA of the clock genes and the Plant dsRNA, with *$p < 0.05$, **$p < 0.01$. Horizontal bars represent day (white filled) and night (black filled) portions of the photo-regime with Zt (Zeitgeber time) indicated below.
