# Supplementary material for: Molecular Oscillator Affects Susceptibility of Caterpillars to Insecticides: Studies on the Egyptian Cotton Leaf Worm—Spodoptera littoralis (Lepidoptera: Noctuidae)
Source: Insects. 2022 May 23;13(5):488. doi: 10.3390/insects13050488 (PMC9147166; doi:10.3390/insects13050488)
Supplement: Supplementary file 1 [file insects-13-00488-s001.zip › Figure S2 ost.pdf]

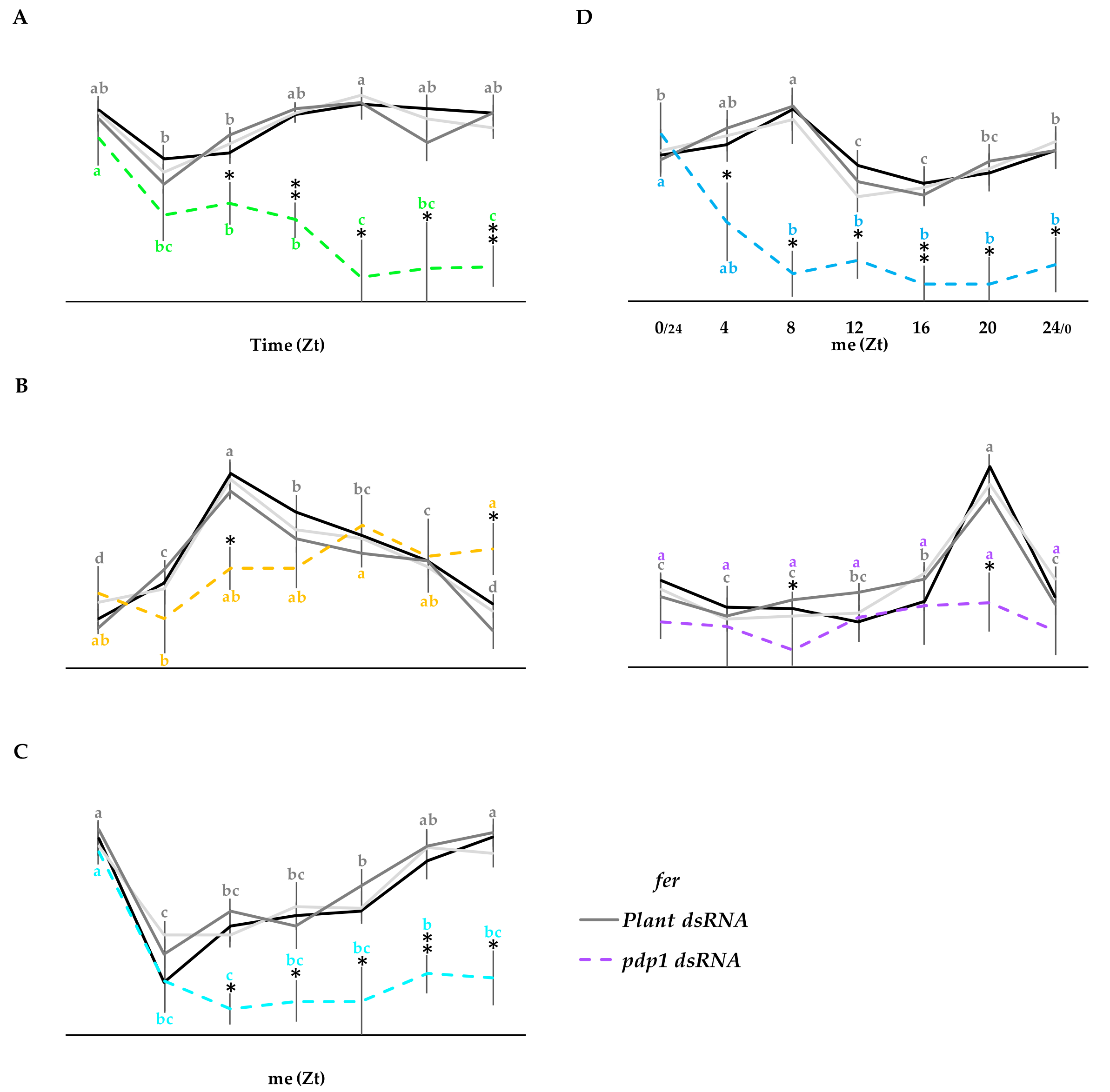

Figure S2. RNAi-mediated disruption of daily patterns of clock genes expression in the midgut. Midguts were isolated from the same animals as the fat bodies and processed using the same methods. Graphs represent daily changes of *per* (A), *tim* (B), *cry2* (C), *cyc* (D), and *pdp1* (E) mRNA levels in larvae treated with dsRNA of clock genes. Statistical analyses of the obtained data and the results presentation in the graphs are analogous to those described in Figure S1.
