# Supplementary material for: Molecular Oscillator Affects Susceptibility of Caterpillars to Insecticides: Studies on the Egyptian Cotton Leaf Worm—Spodoptera littoralis (Lepidoptera: Noctuidae)
Source: Insects. 2022 May 23;13(5):488. doi: 10.3390/insects13050488 (PMC9147166; doi:10.3390/insects13050488)
Supplement: Supplementary file 1 [file insects-13-00488-s001.zip › Figure S3 ost.pdf]

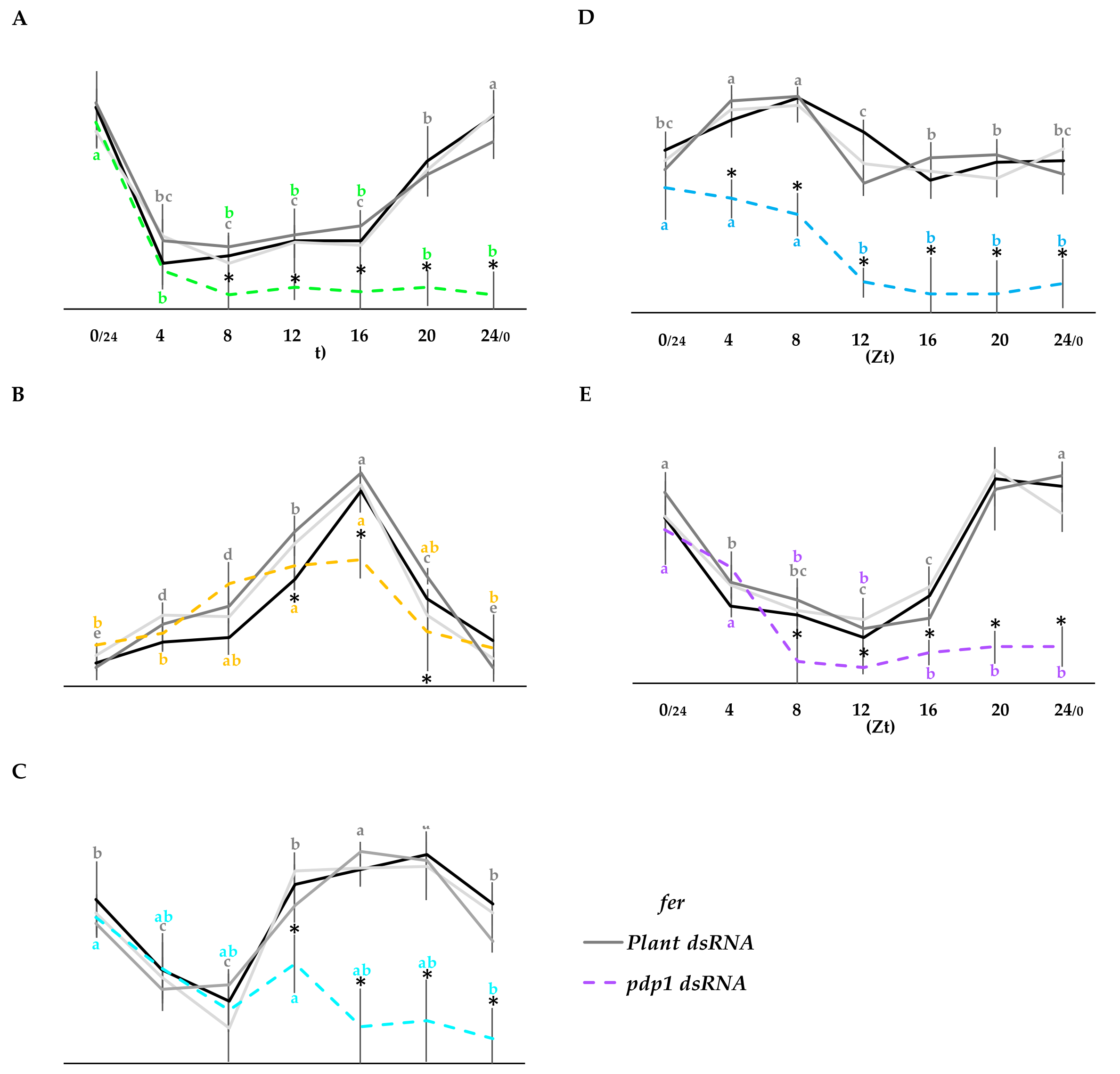

Figure S3. RNAi-mediated disruption of daily patterns of clock genes expression in the Malpighian tubules. Malpighian tubules were isolated from the same animals as the fat bodies and midguts, and processed using the same methods. Graphs represent daily changes of *per* (A), *tim* (B), *cry2* (C), *cyc* (D), and *pdp1* (E) mRNA levels in larvae treated with dsRNA of clock genes. Statistical analyzes of the obtained data and the methods of results presentation on the graphs are analogous to those described on Figure S1.
