# Supplementary material for: Molecular Oscillator Affects Susceptibility of Caterpillars to Insecticides: Studies on the Egyptian Cotton Leaf Worm—Spodoptera littoralis (Lepidoptera: Noctuidae)
Source: Insects. 2022 May 23;13(5):488. doi: 10.3390/insects13050488 (PMC9147166; doi:10.3390/insects13050488)
Supplement: Supplementary file 1 [file insects-13-00488-s001.zip › Table S1 ost.pdf]

Gene	GeneBank accession number	Primers for qPCR		Product size [bp]	Primers for dsRNA synthesis		Product size [bp]
		Sense	Antisense		Sense	Antisens	
per	EF364034.1	CCGAACGAGGTGGTGG TAAA	GCCAAGGTACGGAACA GACT	99	CGCAACACCTTTGCATC ACA	TTTACCACCACCTCGTT CGG	299
tim	FQ029930.1	GAACTGCAAGCAGGCG TCTA	AACTTTGCCGGAGTCC ACAG	94	GGAGACCTGCTTCGCT AAGAT	CTGCGGTCCAGAAATG AGGT	305
cry2	EF396286.1	CAAGTGTATGCGCAAC TCGC	TTCGGACTTGGTGACG ACTG	98	GAGGTGCGATGTGGGT AACT	TGCAGCAGGAACCTCC ATTT	300
cyc	ON052028	GTGGCCAACAACACGA TCGT	ACCTCCCTCTGCTTGTA CATGT	92	GTGGCCAACAACACGA TCGT	TTCTGGTTGAAGACGG CGTC	290
pdp1	ON052027	CCCGCTCGAAGACAAG AAAGAT	ACTTGAGATCGGGGTC ATAGGG	107	TCTCAAGTACGCGGAC CTAGAC	CGGGACTTCTTGATCA TTGGCT	300
EF1-A	HQ177154.1	CGTACTCATTGTCGCC GCTG	CGAGTGTGAAAGCGAG CAGA	95	-	-	-
SSU	M32419.1	-	-	-	GCCTCATTCCCTGTTTC AAG	AGCAAGGAACCCATCC ATTT	292

Table S1. Information about the starters used in the experiments. The primers shown on the two columns on the left (orange) were used to study the daily expression profile of selected clock genes—*period* (*per*), *timeless* (*tim*), *cryptochrome 2* (*cry2*), *cycle* (*cyc*), *pdp1* (*PAR domain protein 1*)—and the *EF1-A* (*Elongation factor 1-alpha*) gene, which was used as a housekeeping standard for normalizing qRT-PCR results. The primers shown on the two columns on the right (purple) were used for *in vitro* synthesis (transcription) to generate dsRNAs used to knock out the expression of the same biological clock genes when injected to the larvae; primers for amplifying *SUU* (*ribulose-1,5-bisphosphate carboxylase small subunit*) of cultivated tobacco were used to produce non-specific (plant) dsRNA which was applied to the larvae in the control group. In addition, the GenBank accession numbers for studied genes and the sizes of products obtained from qRT-PCR and *in vitro* transcription (production of dsRNAs) are shown.
