# Supplementary material for: Molecular Oscillator Affects Susceptibility of Caterpillars to Insecticides: Studies on the Egyptian Cotton Leaf Worm—Spodoptera littoralis (Lepidoptera: Noctuidae)
Source: Insects. 2022 May 23;13(5):488. doi: 10.3390/insects13050488 (PMC9147166; doi:10.3390/insects13050488)
Supplement: Supplementary file 1 [file insects-13-00488-s001.zip › Table S2 ost.pdf]

Evaluated	Group	P-value	F	Robustness (%)	MESOR	Amplitude	Acrophase (º / ZT)
LC50 for fipronil	LD	p<0.05	13.58	90.05	15.98	7.09	103.71/6:54
	LL	p>0.05	0.03	1.97	5.32	0.16	72.73/4:50
LC50 for deltamethrin	LD	p<0.05	9.59	85.20	10.81	11.71	285.56/9:02
	LL	p>0.05	4.46	74.85	6.65	1.74	238.09/15:52
LC50 for malathion	LD	p<0.05	9.75	86.67	24.47	8.76	206.28/13:45
	LL	p>0.05	0.06	3.83	4.44	0.25	16.41/1:05
LC50 for propoxur	LD	p<0.05	10.09	87.19	38.98	11.68	209.83/13:59
	LL	p>0.05	0.03	2.34	6.59	0.43	221.62/14:46
LC50 for acetamiprid	LD	p<0,05	10,42	89,19	8.04	3.19	121,27/8:05
	LL	p>0.05	0.03	2.19	6.29	0.14	31.32/2:05
LC50 for imidacloprid	LD	p<0.05	12.86	88.03	39.78	11.21	86,08/5:44
	LL	p>0.05	1.73	53.53	53.51	6.52	165.49/11:01

Table S2. Daily rhythm analysis of changes in the susceptibility to the six insecticides expressed as LC50 for larvae kept under LD and LL. Data showing the changes of the measured parameter over time were characterised using the cosinor method. According to the rule of cosinor analysis, when the rhythm parameters are taken into account, the values can be mathematically expressed as: $y = M+A(\cos\omega t - \varphi)$. Midline estimating statistics of the rhythm (Mesor – M) is the approximated time series mean of the data for one period, the maximum

value of the approximated best fitting single cosine waveform is the acrophase (φ), the half of the difference between the maximum value and the minimum value of the cosine curve is the amplitude (A), and the time required to complete a cycle is the Period (T). The angular frequency is expressed as $\omega = 2\pi/T$. The mathematical procedure fits the best curve according to the least-squares method. Formulas described by Nelson et al. (1979) and modified by Refinetti et al. (2007) were applied for calculations of A, M, and φ using SigmaPlot 10 (Systat Software, Chicago, IL, USA). The Mesor and rhythm robustness (percentage – percent of total variance accounted by the fit of the single cosine function) were computed and are shown. Rejection with $p < 0.05$ of zero-amplitude assumption of the approximating function was considered to demonstrate significance of rhythmicity. Daily changes in insect susceptibility expressed as LC50 values for insecticides from larvae exposure was considered rhythmic in a statistically significant manner if the percentage of the rhythm was >50% and the $p < 0.05$ for the zero-amplitude test.
