# Supplementary material for: Molecular Oscillator Affects Susceptibility of Caterpillars to Insecticides: Studies on the Egyptian Cotton Leaf Worm—Spodoptera littoralis (Lepidoptera: Noctuidae)
Source: Insects. 2022 May 23;13(5):488. doi: 10.3390/insects13050488 (PMC9147166; doi:10.3390/insects13050488)
Supplement: Supplementary file 1 [file insects-13-00488-s001.zip › Table S3A ost.pdf]

Table S3A. Daily rhythm analysis of *per*, *tim*, *cry2*, *cyc*, and *pdp1* clock gene expressions characterised with qPCR in the fat body of LD reared larvae from Intact (untreated larvae), Buffer (larvae injected with buffer used for *in vitro* transcription–dsRNA production), Plant dsRNA (larvae injected with non-specific dsRNA, that was produced on the sequence of plant gene), with each serving as a control; and one of the clock genes dsRNA—*per*, *tim*, *cry2*, *cyc*, or *pdp1* (larvae injected with dsRNA of the clock gene whose expression was analysed in this particular case). Presented data are based on the calculation of changes in the gene transcript levels in the fat body over time made using the cosinor method, as described in the figure caption for Figure S2. Daily changes in gene expression were considered rhythmic in a statistically significant manner if the percentage of the rhythm was >50% and the p < 0.05 for the zero-amplitude test.

| Organ | Evaluated | Group | P-value | F | Robustness (%) | MESOR | Amplitude | Acrophase (º / ZT) |
|---|---|---|---|---|---|---|---|---|
| fat body | *per* gene expression (mRNA) | Intact | p<0.05 | 12.78 | 89.49 | 70.09 | 23.27 | 277.77/18:31 |
| | | Buffer | p<0.05 | 11.39 | 88.05 | 63.89 | 26.21 | 280.88/18:43 |
| | | Plant dsRNA | p<0.05 | 11.81 | 88.73 | 66.18 | 26.59 | 281.17/18:44 |
| | | *per* dsRNA | p>0.05 | 1.21 | 44.74 | 24.93 | 13.07 | 108.82/7:15 |
| | *tim* gene expression (mRNA) | Intact | p<0.05 | 12.38 | 91.38 | 55.66 | 22.49 | 147.56/9:50 |
| | | Buffer | p<0.05 | 11.26 | 88.44 | 52.28 | 21.11 | 152.55/10:10 |
| | | Plant dsRNA | p<0.05 | 11.08 | 90.89 | 57.01 | 25.37 | 137.72/9:10 |
| | | *tim* dsRNA | p>0.05 | 1.94 | 56.44 | 18.53 | 10.26 | 92.39/6:09 |
| | *cry2* gene expression (mRNA) | Intact | p<0.01 | 128.69 | 98.85 | 64.86 | 31.08 | 292.92/19:31 |
| | | Buffer | p<0.01 | 63.25 | 97.68 | 58.93 | 31.17 | 298.16/19:52 |
| | | Plant dsRNA | p<0,025 | 30.56 | 95.32 | 61.13 | 31.57 | 276.97/18:27 |
| | | *cry2* dsRNA | p>0.05 | 1.76 | 53.99 | 32.77 | 8.01 | 81.46/5:25 |
| | *cyc* gene expression (mRNA) | Intact | p<0.05 | 9.73 | 83.78 | 50.84 | 34.12 | 216.34/14:25 |
| | | Buffer | p<0.05 | 11.65 | 88.59 | 51.08 | 36.85 | 215.86/14:23 |
| | | Plant dsRNA | p<0.05 | 13.29 | 89.68 | 50.72 | 27.43 | 221.89/14:47 |
| | | *cyc* dsRNA | p>0.05 | 0.05 | 3.11 | 15.79 | 1.82 | 209.95/13:59 |
| | *pdp1* gene expression (mRNA) | Intact | p<0.05 | 12.38 | 91.65 | 46.99 | 31.63 | 215.39/14:21 |
| | | Buffer | p<0.05 | 10.57 | 86.45 | 45.74 | 27.55 | 214.14/14:16 |
| | | Plant dsRNA | p<0.05 | 11.05 | 88.05 | 49.35 | 27.71 | 218.99/14:35 |
| | | *pdp1* dsRNA | p>0.05 | 1.09 | 42.20 | 22.37 | 6.69 | 58.37/3:53 |
