# Supplementary material for: Molecular Oscillator Affects Susceptibility of Caterpillars to Insecticides: Studies on the Egyptian Cotton Leaf Worm—Spodoptera littoralis (Lepidoptera: Noctuidae)
Source: Insects. 2022 May 23;13(5):488. doi: 10.3390/insects13050488 (PMC9147166; doi:10.3390/insects13050488)
Supplement: Supplementary file 1 [file insects-13-00488-s001.zip › Table S3B ost.pdf]

Table S3B. Daily rhythm analysis of ECOD, GST, and esterase (xenobiotic metabolizing enzymes) activity in the fat body of LD reared larvae from eight groups—Intact, Buffer, Plant dsRNA, with each serving as a control; and one of five clock genes dsRNA (*per*, *tim*, *cry2*, *cyc* or *pdp1*); group names and description are the same as in the caption for Table S3A. Presented data are based on the calculation of changes in the enzymatic activity in the fat body over time that are made using the cosinor method, as it is described in caption for Figure S2. Daily changes in enzymatic activity was considered rhythmic in a statistically significant manner if the percentage of the rhythm was >50% and the $p < 0.05$ for the zero-amplitude test.

Organ	Evaluated	Group	P-value	F	Robustness (%)	MESOR	Amplitude	Acrophase (º / ZT)
fat body	ECOD activity	Intact	p<0.05	10.33	87.32	0.50	0.24	131.50/8:46
		Buffer	p<0.05	9.85	86.79	0.53	0.30	121.31/8:05
		Plant dsRNA	p<0.025	17.15	92.67	0.53	0.25	119.93/7:59
		per dsRNA	p>0.05	3.72	71.27	0.23	0.05	224.77/14:59
		tim dsRNA	p<0.05	10.35	84.77	0.47	0.18	137.99/9:11
		cry2 dsRNA	p<0.05	11.19	87.58	0.49	0.20	135.87/9:03
		cyc dsRNA	p>0.05	0.52	25.91	0.19	0.045	194.62/12:58
		pdp1 dsRNA	p>0.05	4.90	76.57	0.13	0.03	193.57/12:54
	GST activity	Intact	p<0.05	9.72	88.34	175.21	189.22	272.36/18:09
		Buffer	p<0.05	11.69	88.63	202.76	212.91	274.25/18:17
		Plant dsRNA	p<0.05	11.97	88.86	185.77	214.24	271.77/18:07
		per dsRNA	p>0.05	0.03	2.07	334.65	9.33	256.32/17:05
		tim dsRNA	p<0.05	13.94	78.39	191.04	242.31	274.13/18:16
		cry2 dsRNA	p<0.05	9.97	79.93	309.94	133.69	249.58/16:38
		cyc dsRNA	p>0.05	0.22	12.99	62.35	13.82	199.72/13:18
		pdp1 dsRNA	p>0.05	0.93	38.20	63.26	21.94	342.99/22:51
	esterase activity	Intact	p>0.05	1.11	42.59	50.62	22.65	283.98/18:55
		Buffer	p>0.05	0.46	23.44	53.15	14.41	276.65/18:26
		Plant dsRNA	p>0.05	0.94	38.45	50.25	20.37	280.44/18:41
		per dsRNA	p>0.05	1.01	40.28	93.92	13.19	281.86/18:47
		tim dsRNA	p>0.05	0.36	19.52	82.44	11.15	319.84/21:19
		cry2 dsRNA	p>0.05	1.19	44.23	96.09	11.71	237.55/15:50
		cyc dsRNA	p>0.05	0.01	0.95	30.52	2.75	350.95/23:23
		pdp1 dsRNA	p>0.05	0.04	2.96	31.00	4.96	40.82/2:43
