# Supplementary material for: Molecular Oscillator Affects Susceptibility of Caterpillars to Insecticides: Studies on the Egyptian Cotton Leaf Worm—Spodoptera littoralis (Lepidoptera: Noctuidae)
Source: Insects. 2022 May 23;13(5):488. doi: 10.3390/insects13050488 (PMC9147166; doi:10.3390/insects13050488)
Supplement: Supplementary file 1 [file insects-13-00488-s001.zip › Table S4A ost.pdf]

Table S4A. Daily rhythm analysis of selected clock gene expressions in the larval midgut. The type of data (experimental groups and selected genes), method of analysis, and presentation of results in the table are the same as for Figure S3A.

Organ	Evaluated	Group	P-value	F	Robustness (%)	MESOR	Amplitude	Acrophase (º / ZT)
midgut	*per* gene expression (mRNA)	Intact	p>0.05	4.69	55.29	84.61	13.74	267.61/17:50
		Buffer	p>0.05	3.09	68.09	82.93	15.61	245.89/16:23
		Plant dsRNA	p>0.05	6.39	78.03	81.88	12.89	233.80/15:35
		per dsRNA	p>0.05	5.89	77.37	28.98	19.16	113.49/7:33
	tim gene expression (mRNA)	Intact	p<0.05	9.97	86.97	59.16	25.90	167.99/11:11
		Buffer	p<0.05	9.68	91.05	55.67	24.32	168.58/11:14
		Plant dsRNA	p<0.05	9.96	89.58	53.25	22.71	161.16/10:44
		tim dsRNA	p>0.05	2.44	61.98	49.93	15.08	256.51/17:06
	cry2 gene expression (mRNA)	Intact	p<0.05	9.97	89.49	62.08	22.17	302.12/20:08
		Buffer	p<0.05	10.47	90.89	65.96	20.51	310.29/20:41
		Plant dsRNA	p<0.05	14.85	92.50	68.35	23.36	308.62/20:34
		cry2 dsRNA	p>0.05	7.27	82.89	21.35	8.31	346.05/23:04
	cyc gene expression (mRNA)	Intact	p<0.05	14.50	89.01	70.26	13.98	88.67/5:54
		Buffer	p<0.05	14.78	90.12	68.39	16.68	57.72/3:50
		Plant dsRNA	p<0.05	14.50	90.00	70.48	17.58	70.42/4:41
		cyc dsRNA	p>0.05	1.83	54.91	17.58	10.64	72.44/4:49
	pdp1 gene expression (mRNA)	Intact	p<0.05	11.52	90.32	40.09	25.51	306.29/20:25
		Buffer	p<0.05	14.52	95.11	41.25	27.39	296.76/19:47
		Plant dsRNA	p<0.05	11.71	93.29	41.23	19.66	280.03/18:40
		pdp1 dsRNA	p>0.05	4.58	75.33	21.61	9.43	270.10/18:00
