# Supplementary material for: Molecular Oscillator Affects Susceptibility of Caterpillars to Insecticides: Studies on the Egyptian Cotton Leaf Worm—Spodoptera littoralis (Lepidoptera: Noctuidae)
Source: Insects. 2022 May 23;13(5):488. doi: 10.3390/insects13050488 (PMC9147166; doi:10.3390/insects13050488)
Supplement: Supplementary file 1 [file insects-13-00488-s001.zip › Table S4B ost.pdf]

Table S4B. Daily rhythm analysis of selected xenobiotic metabolizing enzymes in the larval midgut. The type of data (experimental groups and selected enzymes), method of analysis, and presentation of results in the table are the same as for Figure S3B.

Organ	Evaluated	Group	P-value	F	Robustness (%)	MESOR	Amplitude	Acrophase (º / ZT)
midgut	ECOD activity	Intact	p>0.05	0.00007	0.004	22.45	0.20	355.13/23:40
		Buffer	p>0.05	0.0001	0.01	22.48	0.27	19.59/1:18
		Plant dsRNA	p>0.05	0.00007	0.005	22.47	0.20	100.98/6:43
		per dsRNA	p>0.05	0.0000007	0.00005	21.49	0.02	134.75/8:58
		tim dsRNA	p>0.05	0.40	21.27	1.43	0.15	148.76/9:55
		cry2 dsRNA	p>0.05	0.48	24.28	2.73	0.14	143.28/9:33
		cyc dsRNA	p>0.05	2.77	64.88	0.16	0.03	276.87/18:27
		pdp1 dsRNA	p>0.05	0.15	8.94	0.28	0.06	56.99/3:47
	GST activity	Intact	p<0.05	15.05	90.94	273.41	186.98	255.99/17:03
		Buffer	p<0.05	10.50	87.50	303.32	169.32	259.63/17:18
		Plant dsRNA	p<0.05	10.80	87.81	272.78	155.96	257.09/17:08
		per dsRNA	p<0.05	9.77	80.16	484.69	74.17	254.85/16:59
		tim dsRNA	p<0,01	77.56	98.10	306.61	192.44	263.36/17:33
		cry2 dsRNA	p<0.05	10.08	87.04	432.69	172.38	276.41/18:25
		cyc dsRNA	p>0.05	1.93	56.26	101.98	25.01	29.81/1:59
		pdp1 dsRNA	p>0.05	0.88	36.91	59.39	12.42	261.69/17:26
	esterase activity	Intact	p<0.05	9.66	82.94	804.88	317.64	294.16/19:36
		Buffer	p<0.05	9.89	82.19	837.82	205.13	291.75/19:26
		Plant dsRNA	p<0.05	10.49	88.54	770.42	317.86	290.71/19:22
		per dsRNA	p>0.05	1.20	44.46	1314.28	93.13	358.49/23:53
		tim dsRNA	p>0.05	3.60	70.61	1541.98	92.75	164.42/10:57
		cry2 dsRNA	p>0.05	2.62	63.57	1460.79	101.24	353.23/23:32
		cyc dsRNA	p>0.05	0.03	1.73	245.48	22.86	274.82/18:19
		pdp1 dsRNA	p>0.05	9.19	71.01	266.26	116.74	126.12/8:24
