# Supplementary material for: Molecular Oscillator Affects Susceptibility of Caterpillars to Insecticides: Studies on the Egyptian Cotton Leaf Worm—Spodoptera littoralis (Lepidoptera: Noctuidae)
Source: Insects. 2022 May 23;13(5):488. doi: 10.3390/insects13050488 (PMC9147166; doi:10.3390/insects13050488)
Supplement: Supplementary file 1 [file insects-13-00488-s001.zip › Table S6 ost.pdf]

Evaluated	Group	P-value	F	Robustness (%)	MESOR	Amplitude	Acrophase (º / ZT)
LC50 for fipronil	Intact	p<0.05	12.95	89.62	15.84	6.24	106.26/7:05
	Buffer	p<0.05	16.13	94.34	16.92	5.41	104.65/6:58
	Plant dsRNA	p<0.05	15.54	89.69	15.49	6.63	95.15/6:20
	per dsRNA	p>0.05	1.11	42.45	6.20	1.37	352.28/23:29
	tim dsRNA	p<0.05	15.94	91.39	15.41	8.04	104.31/6:57
	cry2 dsRNA	p<0.05	11.51	88.47	17.74	8.78	99.63/6:38
	cyc dsRNA	p>0.05	0.63	29.66	4.36	0.75	306.44/20:25
	pdp1 dsRNA	p>0.05	0.69	31.69	2.72	0.68	297.58/19:50
LC50 for deltamethrin	Intact	p<0.05	13.99	92.68	10.09	10.71	286.74/19:06
	Buffer	p<0.05	14.81	96.25	11.57	12.36	287.63/19:10
	Plant dsRNA	p<0.01	35.09	97.25	11.39	11.81	280.80/18:43
	per dsRNA	p>0.05	0.59	28.39	6.64	1.25	253.31/16:53
	tim dsRNA	p<0.05	14.68	95.75	11.18	12.23	285.66/19:02
	cry2 dsRNA	p<0.05	13.76	91.50	10.84	11.60	287.90/19:11
	cyc dsRNA	p>0.05	0.06	3.96	5.29	0.38	338.59/22:34
	pdp1 dsRNA	p>0.05	9.52	87.52	0.88	0.71	137.44/9:09
LC50 for malathion	Intact	p<0.025	16.87	91.83	24.18	8.11	210.80/14:03
	Buffer	p<0.05	13.57	90.04	25.27	8.72	201.19/13:24
	Plant dsRNA	p<0.05	14.62	95.51	25.21	8.62	216.36/14:25
	per dsRNA	p>0.05	0.11	6.66	2.91	0.26	217.47/14:29
	tim dsRNA	p<0.05	13.93	92.40	23.74	8.01	204.53/13:22
	cry2 dsRNA	p<0.05	19.35	93.33	23.62	8.89	209.81/13:43
	cyc dsRNA	p>0.05	2.51	62.60	2.53	0.70	295.51/19:42
	pdp1 dsRNA	p>0.05	2.99	66.57	2.92	1.23	115.00/7:40
LC50 for propoxur	Intact	p<0.01	65.74	99.28	38.24	11.45	216.48/14:25
	Buffer	p<0.01	34.03	92.88	36.18	12.12	216.37/14:25
	Plant dsRNA	p<0.01	44.83	94.97	34.93	9.69	212.18/14:08
	per dsRNA	p>0.05	0.02	1.28	5.92	0.45	320.36/21:21
	tim dsRNA	p<0.05	14.38	94.49	38.55	10.15	217.52/14:30
	cry2 dsRNA	p<0.05	13.94	92.42	37.42	9.91	220.35/14:41
	cyc dsRNA	p>0.05	0.17	10.27	1.73	0.25	38.12/2:32
	pdp1 dsRNA	p>0.05	1.03	40.77	1.71	0.38	193.29/12:53
LC50 for acetamiprid	Intact	p<0.01	51.42	97.16	7.03	3.16	139.08/9:16
	Buffer	p<0.05	9.98	86.93	7.20	3.50	134.93/8:59
	Plant dsRNA	p<0.025	17.72	92.19	7.35	3.85	131.71/8:46
	per dsRNA	p>0.05	1.46	49.36	38.32	5.35	287.55/19:10
	tim dsRNA	p>0.05	0.72	32.55	44.20	3.55	294.87/19:39

	***cry2* dsRNA**	**p>0.05**	**0.01**	**0.95**	**82.72**	**0.64**	**18.42/1:13**
	***cyc* dsRNA**	**p>0.05**	**0.70**	**31.89**	**2.51**	**0.51**	**220.03/14:40**
	***pdp1* dsRNA**	**p>0.05**	**1.79**	**54.46**	**1.41**	**0.37**	**45.24/3:00**
**LC50 for imidacloprid**	**Intact**	**p<0.05**	**10.24**	**89.97**	**37.45**	**10.85**	**88.23/5:52**
	**Buffer**	**p<0.05**	**10.36**	**89.77**	**38.79**	**12.71**	**91.99/6:07**
	**Plant dsRNA**	**p<0.05**	**10.71**	**71.24**	**38.69**	**14.89**	**85.82/5:43**
	***per* dsRNA**	**p>0.05**	**0.32**	**17.82**	**75.14**	**6.47**	**207.00/13:48**
	***tim* dsRNA**	**p>0.05**	**1.49**	**49.99**	**66.49**	**8.29**	**261.04/17:24**
	***cry2* dsRNA**	**p>0.05**	**0.01**	**0.72**	**131.01**	**0.87**	**309.12/20:36**
	***cyc* dsRNA**	**p>0.05**	**0.86**	**36.34**	**13.75**	**0.63**	**262.75/17:31**
	***pdp1* dsRNA**	**p>0.05**	**0.55**	**26.99**	**3.09**	**0.32**	**338.73/22:34**

Table S6. Daily rhythm analysis of changes in the susceptibility to six insecticides expressed as LC50 for larvae with altered activity of the molecular oscillator using the RNAi technique and kept in LD 16:8. For each insecticide, the effects of Intact, Buffer and Plant dsRNA (each served as a control) and one of five clock genes dsRNA (*per*, *tim*, *cry2*, *cyc* or *pdp1*) were analysed; group names and description are the same as in the caption for Table S3A. Presented data are based on the calculation of changes in the LC50 values over time that are made using the cosinor method, as it is described in caption for Figure S2. Daily changes in the LC50 for insects of each group (controls or injected with dsRNA) treated with particular insecticides were considered rhythmic in a statistically significant manner if the percentage of the rhythm was >50% and the $p < 0.05$ for the zero-amplitude test.
