# Supplementary material for: Molecular Oscillator Affects Susceptibility of Caterpillars to Insecticides: Studies on the Egyptian Cotton Leaf Worm—Spodoptera littoralis (Lepidoptera: Noctuidae)
Source: Insects. 2022 May 23;13(5):488. doi: 10.3390/insects13050488 (PMC9147166; doi:10.3390/insects13050488)
Supplement: Supplementary file 1 [file insects-13-00488-s001.zip › Text file S1 ost.pdf]

Supplementary methods

i. Quantitative real time PCR (qPCR)

Levels of *per*, *tim*, *cry2*, *cyc*, *vri*, and *pdp1* mRNA were measured using total RNA extracted from the collected organs according to the Renozol RNA (GenoPlast Biochemicals, Rokocin, Poland) and treated with DNase I – RNase-free (New England BioLabs – NEB Inc., Ipswich, MA, USA). Equal amounts of RNA were pooled from samples obtained from 10 to 12 separate larvae at each time point and converted to cDNA using First Strand cDNA PLUS Synthesis Kit (GeneOn GmbH, Ludwigshafen, Germany) with Oligo-$(dT)_{20}$ primers. Gene-expression profiles were generated using Bio-Star qPCR-Mastermix SYBRGreen HIGH-ROX (GeneOn GmbH) and gene specific primers (Genomed S. A., Warsaw, Poland) (Table S1) in the StepOnePlus Real-Time PCR System (Applied Biosystems, Waltham, MA, USA). Relative transcript abundance was calculated as follows: each Ct was subtracted from the lowest Ct on the same assay plate to obtain a relative Ct (relCt) value. PCR efficiency (Ef) was calculated for each reaction using QuanSoft software, and the normalization factor (Nf) was determined for each sample by GeNorm algorithm (Biogazelle NV, Zwijnaarde, Belgium), with *Elongation Factor 1-Alpha (EF1-A) gene* chosen as a housekeeping reference gene. All reactions were performed in triplicate. An efficiency-corrected, normalized, relative gene-expression value (Ef^{relCt}/Nf) was used to calculate the mean of each reaction triplicate. The highest relative expression level was set to 100 for each bio-repeat and statistical significance (at $p < 0.05$) was determined by one-way ANOVA followed by Bonferroni's post-hoc test.

ii. cloning of *pdp1* and *cyc* cDNA from *S. littoralis*

The published sequences of PAR-domain protein 1 (PDP1) from *Helicoverpa armigera*, *Ostrinia furnacalis*, and *Danaus plexippus* (GenePept: AYA44273.1, GenePept: AGR44476.1, and GenePept: ABV22507.1, respectively); and hepatic leukemia factor-like from *Spodoptera frugiperda*, *Spodoptera litura*, *Trichoplusia ni*, and *Bombyx mandarina* (GenPept: XP_035439260.1, GenPept: XP_022832420.1, XP_026742228.1, and XP_028026836.1, respectively) were compared for conserved regions using ClustalW software [22]. The gene sequences of protein cycle (CYC) from *S. litura*, *S. frugiperda*, *Manduca sexta*, *H. armigera*, and *T. ni* (GenePept: XP_022833214.1, GenePept: XM_035583465.1, GenePept: XP_037299673.1, GenePept: XP_021194690.1, and GenePept: XP_026742235.1, respectively) and Aryl Hydrocarbon Receptor Nuclear Translocator-like Protein 1 from *Galleria mellonella* (GenePept: XP_031767216.1) were aligned and compared in the same manner; these regions were used to design degenerate primers. A set of primers SLpdp1-degF2, 5'- CCNGTCYMSTGTNGTTGTATC -3' and SLpdp1-degR2, 5'-CTTMAGYAGCTCSAVTHCTTGTC -3' for *S. littoralis pdp1*, and SLcyc-degF1, 5'-AAGTNCCTYTTCGTCGNCCAG -3' and SLcyc-degR1, 5'-CAGSAYGTTKGCTTTNAGTGG -3' for *S. littoralis cyc* (Genomed S. A.), resulted in amplification of the expected bands. Total RNA from Malpighian tubules of 2-day old last instar larvae of *S. littoralis* (collected at different times of the day) was extracted with Renozol RNA (GenoPlast Biochemicals) and treated with DNase I–RNase-free (NEB Inc.). First strand synthesis was performed with AMV Reverse Transcriptase (NEB Inc.), using 5 pmol of Oligo-$(dT)_{18}$ Primer (NEB Inc.) and 15 μg of total RNA.

Touchdown PCR was performed in an MJ Research PTC-200 Gradient Thermal Cycler (MJ Research, Inc., Waltham, MA, USA) under the following conditions: 94 °C for 5 min followed by 10 cycles at 94°C for 1 min, 41 °C for 2 min with ramp 7 °C/min to 72 °C and 3 min at 72 °C, then 20 cycles at 94°C for 1 min, 41°C for 2 min, and 72 °C followed by a final extension at 72 °C for 15 min. The generated PCR fragments ~730 bp (for *pdp1*) and ~520 bp

(for *cyc*) were gel purified and ligated into the pCR II TOPO vector using the pCR TOPO® TA cloning kit (Thermo Fisher Scientific, Waltham, MA, USA). Both sequences were determined by sequencing with M13-reverse primer (Genomed S. A.) and compared for similarity to *pdp1* and *cyc* coding sequences from other lepidopteran species using BLAST. The sequences were submitted (Genbank: ON052027 for *pdp1* and ON052028 for *cyc*) and used to design specific primers for analysis of *pdp1* and *cyc* genes expression in different tissues and for dsRNA production of *S. littoralis*.
